# Human Olfactory Mucosa Stem Cells Delivery Using a Collagen Hydrogel: As a Potential Candidate for Bone Tissue Engineering

**DOI:** 10.3390/ma14143909

**Published:** 2021-07-13

**Authors:** Sara Simorgh, Peiman Brouki Milan, Maryam Saadatmand, Zohreh Bagher, Mazaher Gholipourmalekabadi, Rafieh Alizadeh, Ahmad Hivechi, Zohreh Arabpour, Masoud Hamidi, Cédric Delattre

**Affiliations:** 1 Cellular and Molecular Research Centre, Iran University of Medical Sciences, Tehran 1591639675, Iran; srsimorgh@gmail.com (S.S.); mazaher.gholipour@gmail.com (M.G.); ahmadhivechi2020@gmail.com (A.H.); 2 Department of Tissue Engineering and Regenerative Medicine, Faculty of Advanced Technologies in Medicine, Iran University of Medical Sciences, Tehran 1591639675, Iran; Baharebagher@gmail.com; 3 Department of Chemical and Petroleum Engineering, Sharif University of Technology, Tehran 111559465, Iran; m.saadatmand@sharif.edu; 4 ENT and Head and Neck Research Center and Department, Hazrat Rasoul Akram Hospital, The Five Senses Health Institute, Iran University of Medical Sciences, Tehran 1445613111, Iran; nafiseh.alizadeh@gmail.com; 5 Department of Tissue Engineering and Applied Cell Sciences, School of Advanced Technologies in Medicine, Tehran University of Medical Sciences, Tehran 1417613151, Iran; arabpourzohreh@gmail.com; 6 Iranian Tissue Bank & Research Center, Tehran University of Medical Sciences, Teheran 1419733141, Iran; 7 Department of Medical Biotechnology, Faculty of Paramedicine, Guilan University of Medical Sciences, Rasht 4477166595, Iran; Masoud.Hamidi@ulb.be; 8 Université Clermont Auvergne, Clermont Auvergne INP, CNRS, Institut Pascal, F-63000 Clermont-Ferrand, France; 9 Institut Universitaire de France (IUF), 1 Rue Descartes, 75005 Paris, France

**Keywords:** tissue engineering, collagen hydrogel, cell delivery, bone regeneration, olfactory ectomesenchyme stem cells

## Abstract

For bone tissue engineering, stem cell-based therapy has become a promising option. Recently, cell transplantation supported by polymeric carriers has been increasingly evaluated. Herein, we encapsulated human olfactory ectomesenchymal stem cells (OE-MSC) in the collagen hydrogel system, and their osteogenic potential was assessed in vitro and in vivo conditions. Collagen type I was composed of four different concentrations of (4 mg/mL, 5 mg/mL, 6 mg/mL, 7 mg/mL). SDS-Page, FTIR, rheologic test, resazurin assay, live/dead assay, and SEM were used to characterize collagen hydrogels. OE-MSCs encapsulated in the optimum concentration of collagen hydrogel and transplanted in rat calvarial defects. The tissue samples were harvested after 4- and 8-weeks post-transplantation and assessed by optical imaging, micro CT, and H&E staining methods. The highest porosity and biocompatibility were confirmed in all scaffolds. The collagen hydrogel with 7 mg/mL concentration was presented as optimal mechanical properties close to the naïve bone. Furthermore, the same concentration illustrated high osteogenic differentiation confirmed by real-time PCR and alizarin red S methods. Bone healing has significantly occurred in defects treated with OE-MSCs encapsulated hydrogels in vivo. As a result, OE-MSCs with suitable carriers could be used as an appropriate cell source to address clinical bone complications.

## 1. Introduction

Bone defects represent a primary concern of disability worldwide [1]. Although there have been enormous advances in developing new drugs, surgical methods and treatment modalities, clinical outcomes remain fundamentally undesirable in many patients. Autologous bone grafting has been popularized as a therapeutic tool, accelerating the overgrowth of cells that can regenerate new bone tissue [2]. Restricted access to bone tissue has been widely challenged in the clinic [3]. Hence, developing new strategies for bone regeneration is highly required. As a new area with a clinical prospect, tissue engineering has emerged as powerful platforms for tissue reconstruction [4].

Biomaterials as a main component of tissue engineering have emerged as effective cell delivery systems to accelerate their survival and proliferation rate and differentiation capacity [5]. Up to now, many types of polymeric materials and bone substitutes have been studied and developed to regenerate bone defects. Among them, hydrogels contain hydrophilic cross-linked networks with water-swelling capacity have a great potential to increase cell adhesion and migration with biodegradation property [6]. Therefore, these bioactive three-dimensional scaffolds provide appropriate conditions for cell proliferation and differentiation. Living cells can be encapsulated in hydrogels as a cell delivery system and can be easily transferred to the target region for tissue regeneration [7].

Collagen is one of the most important structural proteins in the extracellular matrix (ECM). A large and growing body of literature has emphasized the application of collagen in medicine owing to its supreme biocompatibility and low immunogenicity [8]. Since bone ECM is composed of hydroxyapatite mineral deposits in collagen type I substrate, collagen hydrogel can be a suitable candidate for stem cell encapsulation with flexibility analogous to natural tissue features for bone regeneration [9]. Additionally, the different type of stem cell has been examined in the tissue engineering field and can be isolated from adult and embryonic tissues [10]. Immunomodulatory effects, releasing paracrine molecules, and remarkable angiogenic capacity are known as the leading mechanisms for tissue regeneration [11]. Various mesenchymal stem cells (MSC) sources, such as dental pulp MSCs, human amniotic MSCs, placental-derived MSCs, and mesenchymal cell derived from bone marrow and adipose tissue, have been identified for bone regeneration [12]. Clinical trials of autologous adult stem cells transplantation for bone repair exhibited long term safety and efficacy [13]. Researchers have shown a growing trend towards using olfactory-derived ectomesenchymal stem cells (OE-MSC) as an appropriate available stem cell source for therapeutic strategies [14,15,16]. Human olfactory mucosa consists of two layers, lamina propria, and the epithelial layer. The globose and basal cells lay quiescent in the epidermal layer of olfactory mucosa, involving epithelial regeneration. The olfactory mucosa also contains multipotent cells in the lamina propria with neuronal and mesenchymal properties. These stem cells have been considered as a new family of MSCs due to their easy accessibility and autologous grafting ability [17,18] to solve immune system rejection and ethical issues in clinical trials [19].

There has been exciting research that recognized the critical immunomodulatory role played by olfactory-derived stem cells via releasing TGF-B and IL10 [20]. Moreover, it has been shown that OE-MSCs have a higher differentiation capacity into osteocyte than progenitor cells found in the umbilical cord [15,21]. OE-MSCs have been successfully used for nerve regeneration [22,23]. Due to its capacity to osteocyte [17], It can be appropriate sources for in vivo study of bone regeneration. However, no comprehensive research has been performed on OE-MSCs and their application in vivo condition for bone tissue engineering.

The main questions have been raised about the behavior of human OE-MSCs in an osteogenic microenvironment. Accordingly, in this research, we encapsulated human OE-MSCs in a collagen hydrogel system, and their osteogenic potential was evaluated in vitro conditions. After optimization of collagen concentration, we transplanted this system to assess in vivo bone condition in a rat calvaria defection.

## 2. Materials and Methods

### 2.1. Materials

Ethylenediaminetetraacetic acid (EDTA) was purchased from Gibco, Canada Inc. Ethanol, methanol, and acetic acid (100%) were obtained from Merck, Germany. Polyacrylamide, Alizarin Red, Dil, The fetal bovine serum was given by Gibco (FBS). Primers and cDNA synthesis kit were obtained from Sinaclon, IRAN. B-glycerophosphate and dexamethasone were prepared from SANTA CRUZ biotechnology. The SYBR-Green PCR Master Mix was given by ThermoFisher Scientific in the United Kingdom.

### 2.2. Ethics

This investigation was confirmed by IUMS Ethical Committee (NO; IR.IUMS.REC.1399.020). All procedure performed according to the University guidelines.

### 2.3. Experimental Studies

#### 2.3.1. Isolation and Characterization of Cells

Olfactory mucosa samples were harvested from human sources and processed, as previously defined [1]. Shortly, the tissue pieces were minced and transferred to a sterile dish, and OE-MSCs isolation was carried out as previously reported [2]. The isolated cells were cultured in DMEM/F12 nutrient mixture and proliferated. After that, cells were characterized by flow cytometry assessment for specific proteins like CD90, CD29, CD105, CD31, and CD34. The adipogenic and osteogenic capacity of stem cells were assessed on day 21. Moreover, to detect mesenchymal cells markers, immunocytochemistry was done, and the expression of nestin and vimentin were assessed.

#### 2.3.2. Collagen Extraction, Hydrogel Preparation, and Characterization

##### Rat Tail Collagen Type I Extraction

First of all, we harvested tendons from the rat tails [3]. Tissue samples transferred to a petri dish containing 70% ethanol. The tendons then were separated from the sheath attached to the vertebrae. The extracted tendons were transferred in 0.02% acetic acid under moderate vibration at 4 °C. After that, the solution was centrifuged for 1 h at 12,000× *g* and deposited at −80 °C. The frozen solution was transferred to a freeze dryer machine to obtain dried collagen.

##### Fourier Transform Infrared Spectroscopy (FTIR)

The detection of amide bands was done using Fourier transform infrared spectroscopy (FTIR) [4]. The spectra were collected in a Nicolet 6700 spectrometer (Mundelein, IL, USA) and documented with a resolution of 4 cm in 1 over 64 scans.

##### Gel Electrophoresis

Gel electrophoresis sodium dodecyl sulfate-polyacrylamide (SDS-PAGE) was carried out to detect collagen type I according to previous studies [5]. Gel electrophoresis was prepared by a combination of running and stacking gels. After electrophoresis, the gels were set and dyed in Coomassie Blue R-250 (0.1%) for four hours at 60 °C in a 25% solution; then several rinses in a decolorizer solution containing methanol, acetic acid, and water (2:3:35, V/V) dissolved the residual stain.

##### Hydrogel Preparation and OE-MSCs Encapsulation

Collagen type I extracted from rat tail was dissolved in 1 mL acetic acid 2 N. This solution was mixed with phosphate buffer saline (10 × PBS) and NaOH (1N) at pH 7.4 while kept on ice [6] and was prepared in four final separate concentrations including 4 mg/mL, 5 mg/mL, 6 mg/mL, and 7 mg/mL. The human OE-MSCs pellet was resuspended in cell culture medium and 1 × 10^4^ cells/mL mixed into the non-gelated collagen mixture, followed by immediate gelation in 37 °C incubation, and the cell culture medium was added on top of the gelated hydrogels [7].

##### Field Emission Scanning Electron Microscopy

The morphology and elemental composition of the specimens were determined using digital field emission scanning electron microscopy [8]. OE-MSCs had been cultured separately on four concentrations of hydrogels (4 mg/mL, 5 mg/mL, 6 mg/mL, and 7 mg/mL) for 48 h; until drying with a critical point dryer, the encapsulated OE-MSCs in four concentrations of collagen hydrogels is dehydrated in a graded sequence of ethanol concentrations. Gold was sputtered on the dried specimens. The spectrum was mapped and analyzed using energy dispersive spectroscopy (TESCAN CLARA, Brno-Kohoutovice, Czechia).

##### Porosity Assessment

To determine porosity of scaffolds, the liquid displacement method was done [9]. Ethanol was applied for liquid displacement because it did not cause shrinking or swelling of the scaffold. The scaffolds were weighed, soaked in ethanol for 30 min, and then the weight of the scaffolds in ethanol [10] was determined. The filter paper was used to extract the ethanol from the scaffold base, and the weight of the wet scaffold (ww) was also calculated (*n* = 3). The scaffold porosity was estimated by Equation:

Porosity (%) = (ww − wd)/(ww − wl) × 100

##### Rheological Analysis

The effect of concentration on the storage (G′) and loss modulus (G″) of collagen hydrogels was evaluated by rheometric mechanical spectroscopy (RMS) tests using an MCR300 rheometer (Anton Paar, Graz, Austria) equipped with a cone-plate geometry [11]. For sample preparation, the hydrogels were poured into a mold and then placed on the plate of the rheometer; and before beginning the procedure, surface water was carefully removed with tissue paper. Frequency sweeps were performed at frequencies ranging from 0.01 to 100 Hz. At a stable temperature of 37 °C, both tests were carried out.

##### OE-MSCs Viability and Proliferation Assessment

The viability of human OE-MSCs was quantified using a live/dead viability package (ThermoFisher, Berlin, Germany), following the manufacturer’s staining protocol [11]. Briefly, after stem cell encapsulation in collagen hydrogels on days 1, 7, and 14, the samples were dyed for 10 min after the culture medium was extracted. The cells were then washed with PBS and observed under the fluorescence microscope. By staining the hydrogels with fluorescent diacetate (FDA) and propidium iodide (PI), the existence of encapsulated OE-MSCs was determined. FDA indicates living cells with green fluorescence, while PI indicates dead cells with red fluorescence. Furthermore, for proliferation quantitative evaluation, resazurin kit (Kiazist, Tehran, Iran) was used at various time points (1st, 7th, and 14th days after stem cell encapsulation). Following the removal of the media, 10 mL of resazurin reagent containing 180 mL of serum-free medium was applied to each well of a 96-well plate and incubated for 4 h at 37 °C. Using a microplate reader, the absorbance values of each well were calculated at 570 nm.

##### Osteogenic Differentiation

Encapsulated OE-MSCs were included in collagen hydrogels containing 10% minimum essential medication eagle alpha modification (Alpha MEM) supplemented with fetal bovine serum, 1% PS, 50 g/mL L-ascorbic acid, 100 nM dexamethasone, and 10 mM b-glycerophosphate in the osteogenic separation media [12]. For two to three weeks, the culture medium was updated twice a week.

##### ECM Mineralization

Alizarin Red S staining as a quantitative and qualitative evaluation was done to extracellular matrix mineralization study on day 21. The samples were set in 10% formalin at room temperature before being stained with Alizarin Red (catalogue N 5533-25G, Sigma–Aldrich) for 30 min at a pH of 4.1 to 4.5. The samples were then washed three times with purified water and photographed under a microscope in each well. The 10% acetic acid was applied to samples and incubated for 30 min at room temperature to determine calcium content. After being heated for 10 min at 85 °C, the sample was put on ice for 5 min before being centrifuged at 20,000 g for 15 min. A total of 200 μL of supernatant is combined with 75 μL of 10% ammonium hydroxide. Alizarin red staining was assessed using a plate reader (BioTek, Oxfordshire, UK) set to 405 nm and light microscopic images [13].

##### Gene Expression of Bone Markers

On days 14 and 21, gene expression for the osteogenic markers runt-related transcription factor 2 (Runx2), collagen type I (Col 1), osteopontin (OPN), osteocalcin [14], and alkaline phosphatase was determined using real-time PCR. According to manufacturer protocol [15], max RNA was collected from the samples of five classes using an RNX-plus solution (PRIMA Scientific Co, Favorgen, Thai). Then, using oligo [16] primers and a cDNA synthesis package (QIAGEN-BIOTECH, Hilden, Germany), complete RNA was transformed to cDNA, and real-time RT-PCR analysis was performed using PCR master mix green-high RoxA325402, and the operation was built using the ABI step one unit (Applied Biosystems, Sequences Detection Systems, Foster City, CA, USA) for 40 cycles.

### 2.4. In Vivo Study

#### 2.4.1. Calvarial Defect Model

Parietal calvarial defects were made in two-month-old male rats with an average weight of 200 to 250 g. The animals were anaesthetized by intramuscular injection of ketamine hydrochloride (35 mg/kg) and xylazine (2 mg/kg). To expose the parietal bone, a break was made just outside the sagittal midline. To avoid dora perforation, the pericranium was cut, and a 7 mm defect on the calvaria was produced with a trifoliate drill [17]. The surgical site was cleaned with a saline solution. The rats were randomly divided into three groups (*n =* 5) as follows: group 1: calvarial defection without any treatment as a control group; group 2: calvarial defection treated by 7 mg/mL collagen hydrogel (blank scaffold); and group 3: calvarial defection treated by OE-MSCs that encapsulated in 7 mg/mL collagen hydrogel (OE-MSCs).

#### 2.4.2. OE-MSCs Labelling and Implantation

Dil dye was used as a fluorescent lipophilic cationic carbocyanine to tag OE-MSCs membranes. According to the protocol mentioned in a previous study [18,19], OE-MSCs were tagged with 4 μg/mL Dil for 30 min at 37 °C and then OE-MSCs were washed twice with PBS. Each defect in the Hydrogel+OE-MSCs group was filled with 1 × 10^6^ OE-MSCs encapsulated in 1 mL of 7 mg/mL collagen hydrogel. The skin was sutured after implantation.

#### 2.4.3. Optical Imaging

DiI-labelled human OE-MSCs tracing was performed to evaluate the survival of OE-MSCs after 72 h in rat calvaria defection [20]. Therefore, 72 h after transplantation, optical images were taken by Kodak FX (Connecticut 06511, USA) pro system to stem cell tracing.

#### 2.4.4. Micro-Computed Tomography

Four and eight weeks after surgical implantation, samples were taken. At any time point, the animals were sacrificed by CO_2_ asphyxiation with cervical dislocation. The calvaria of the skull was removed and retained in 10% formalin. The reconstructed cranium morphology was assessed using a micro-CT system (LOTUS-NDT, Behin Negareh Co., Tehran, Iran). The voltage and current of the X-ray tube are set to 80 kV and 85 A, respectively. In this analysis, no additional filtration was used. The scan took about 2 h, with a nominal resolution of 10 microns. The LOTUS NDT-ACQ program handled both protocol configurations. LOTUS NDT-REC was used to reconstruct the acquired 3D data using a simple Feldkamp, Davis, Kress (FDK) algorithm [21]. On the defect field, a 7 mm diameter volume of interest (VOI) was chosen. Then, because the sample had soft tissue and different parts, thresholding was used for the segmentation of VOI into other parts. Then, by calculating the number of voxels related to newly formed bone, bone volume (BV) was obtained, and the amount of bone volume fraction in VOI was calculated with BV/TV. TV: total volume of regenerated bone, pores, and soft tissue trapped in analyzed 3D volume of interest (VOI) selected on structures (unit: mm^3^). BV: volume of regenerated bone-in analyzed 3D volume of interest (VOI) selected on structures (unit: mm^3^). Bone volume fraction: The fraction of BV/TV (unit: percent).

#### 2.4.5. Hematoxylin and Eosin (H&E) Staining

In order to evaluate bone formation, H&E staining was carried out [22]. After fixing the samples in 4% paraformaldehyde, After decalcification, the tissues were dehydrated in an ascending sequence of ethanol and then placed in paraffin. Sections that were 5 m thick were prepared and stained with (H&E). The reconstructed bone tissue photograph obtained under a microscope (ZEISS Axio Scope A1, Oberkochen, Germany) was quantified using ImageJ software.

### 2.5. Statistical Evaluation

The statistical analysis in this article was performed using the Graph Pad PRISM 7.03 software. All findings were evaluated using Student’s t-tests for two comparisons and one-way ANOVA analysis of variance followed by Tukey’s test. Both statistical data are shown using the mean and standard deviation of the mean. The statistical significance level was set at *p* ≤ 0.05.

## 3. Results

### 3.1. Collagen Type I Extracted from Rat Tail and Characterized Next Prepared Collagen Hydrogel

**Figure 1:**
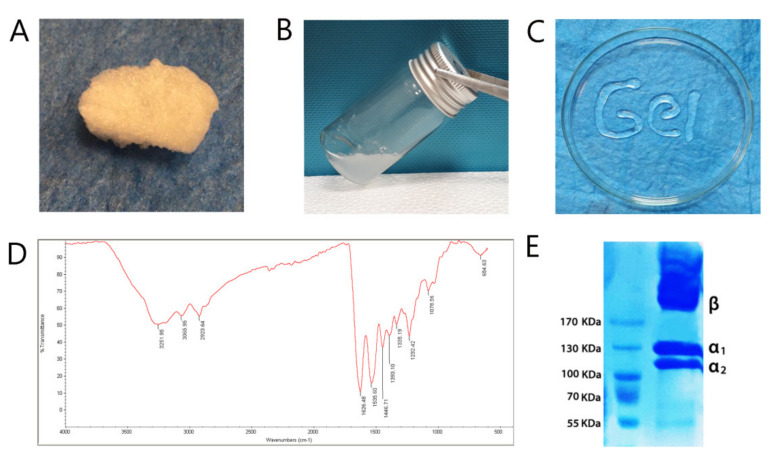
Characterization of collagen type I, which is extracted from rat tail. (**A**) The freeze-dried collagen; (**B**) the collagen solution at 7 mg/mL concentration; (**C**) self-assembled collagen, in pH 7.4 and 37 °C was injected via a 0.7 mm diameter syringe; (**D**) The FTIR spectrum shows the presence of specific molecular vibrations such as amide A, amide B, amide I, amide II, and amide III, which certified the attendance of type 1 collagen structure; (**E**) The inclusion of monomer α_1_, α_2_, and β dimers and dimers in sodium dodecyl sulfate-polyacrylamide electrophoresis data revealed collagen type I structure. In addition, the same molecular weight (MW) was found between the collagens.

Collagen type I was lyophilized and dissolved at a concentration of 7 mg/mL in acetic acid. The self-assembly of collagen was done in pH 7.4 and 37 °C. The hydrogel was injected easily and continually via a 0.7 mm diameter syringe (Figure 1A–C).

The spectrum in FTIR showed the absorption peaks at 3251.95 cm^−1^ is associated with –OH. The band at 2923.64 cm^−1^ has corresponded with CH3 and CH2 symmetric stretching, respectively. At 1446.71 cm^−1^, the C-N deformation reached its maximum. The bands at 1626.48 cm^−1^, 1535.60 cm^−1^, and 1232.42 cm^−1^, respectively, reflect Amide I (C=O), Amide II (N-H stretching and C-N deformation), and Amide III (C-N deformation and N-H stretching) (Figure 1D). SDS-PAGE was carried out to certify the type of collagen extracted from the rat tail. The protein ingredients were isolated according to their electrophoresis volubility (Figure 1E). The extracted collagen showed two distinct bands that are attributed to two different types of α chain and β-chains. The molecular weight of α_1_ collagen type I was about 125 kDa, while α_2_ was approximately 110 kDa, and β-chains was higher than 170 kDa. Additionally, the ratio of the α_1_ band was higher than the α_2_ band (Figure 1E).

### 3.2. OE-MSCs Isolated from Human Olfactory Mucosa and Characterized

**Figure 2:**
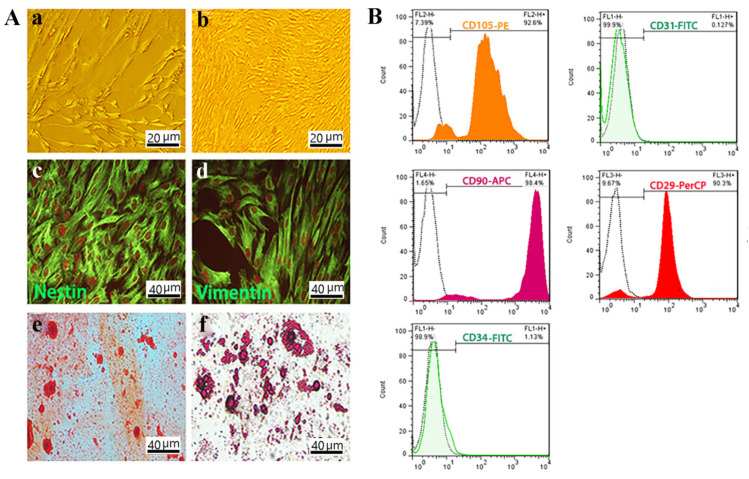
OE-MSCs characterization. (**A**) Morphology of OE-MSCs in passage one. Scale bar: 20 μm; (**a**,**b**) Morphology of OE-MSCs in passage three which was described as a spindle-like morphology. Scale bar: 10 μm; Immunocytochemistry of OE-MSCs to characterized specific neural crest and mesenchymal markers; (**c**) nestin; and (**d**) vimentin by immunofluorescence staining. Osteogenic and adipogenic differentiation of OE-MSCs, respectively; (**e**,**f**) Scale bar: 40 μm. (**B**) Flow cytometric assessment to characterization of OE-MSCs surface markers; including CD29 as a positive marker; CD31 as a negative marker; CD105 as a positive marker; CD90 as a positive marker; and CD34 as a negative marker.

Based on the images obtained from the optical microscope, the morphology of OE-MSCs isolated from human olfactory mucosa appears to be completely homogeneous. It resembles the spindle-like mesenchymal cells (Figure 2A). Furthermore, immunocytochemistry analysis showed the significant expression of nestin and vimentin as neuronal and mesenchymal markers, respectively (Figure 2A).

Besides, the differentiation of human OE-MSCs into osteoblasts and adipocytes was assessed by Alizarin red S and Oil red O staining, respectively, with the results suggesting high mineralization and high adipogenic potency of OE-MSCs (Figure 2A).

The OE-MSCs is positive for CD90, CD29, and CD105 in greater than 98.4%, 90.3%, and 92.6% of the time, respectively, according to flow cytometry study as MSCs special surface markers. In addition, these cells were negative for CD31 and CD34, respectively, 0.127% and 1.13% (Figure 2B).

### 3.3. The Increase in the Concentration of Collagen Leads to Enhance Stem Cell Viability and Proliferation of OE-MSCs

**Figure 3:**
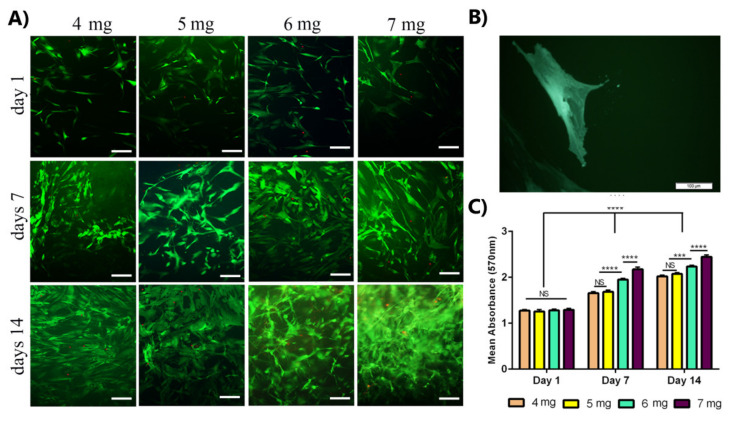
Evaluation of human OE-MSCs viability that encapsulated in different concentrations of collagen hydrogels for 14 days. (**A**) Live/dead fluorescence images of OE-MSCs cultured in collagen hydrogels, the green color shows the living cells, and the red color shows the dead cells (scale bar = 100 µm); (**B**) Images of OE-MSC in the bulk of collagen hydrogel (scale bar = 100 µm); (**C**) Comparison of the proliferative activity of OE-MSCs encapsulated in different concentrations of collagen hydrogels by resazurin assay (NS, no significant difference; *p* < 0.05, *** *p* < 0.001, **** *p* < 0.0001, the Scale Bars: 100 μm).

Based on the images obtained from the live/dead assay (Figure 3A), the OE-MSCs attachment and proliferation with elongated (green) morphology in collagen hydrogel were evident in all samples. Cell proliferation was related to collagen concentration and cell culture time. On day 14, the highest cell proliferation and survival were observed in the collagen 7 mg/mL group, and cell density was higher on day 7 than on day 1. In comparison, the highest density was shown on day 14. Only a small number of dead (red) cells were visible in all samples.

Metabolic and proliferative activity of OE-MSCs in collagen hydrogel was evaluated using the resazurin method at different time points (1, 7, and 14 days after cell culture). According to the results, as shown in Figure 3B, proliferative activity depended on collagen concentration and cell culture time for all samples. The metabolic activity was higher on day 14 than on other days. Comparing the results for different concentrations of collagen showed that the proliferative activity of human OE-MSCs was increased on days 7 and 14 with increasing collagen concentration, and on days 7 and 14, there was a significant difference between 7 mg/mL and 6 mg/mL groups. The proliferative activity of OE-MSCs encapsulated in collagen 7 mg/mL was higher than other groups at every time points.

### 3.4. Hydrogel Properties and Biocompatibility

**Figure 4:**
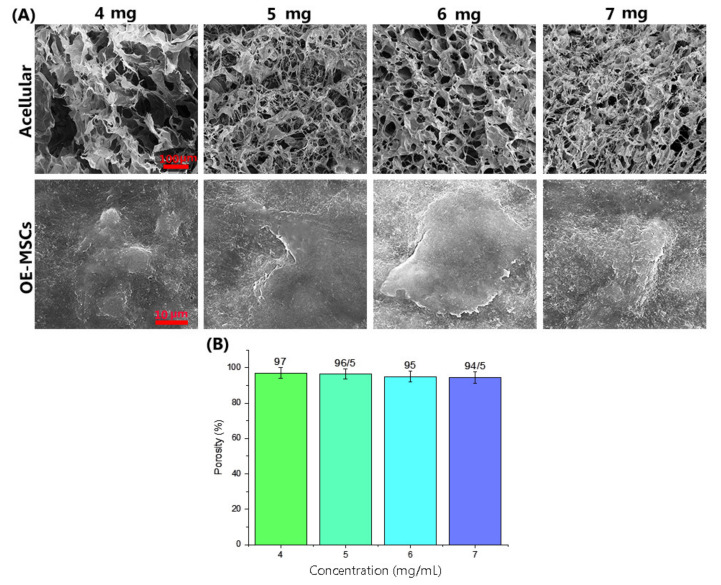
(**A**) FE-SEM images of the collagen hydrogel structures with different concentration of collagen (Scale bar: 100 μm) and OE-MSCs encapsulated in different concentration of collagen hydrogel (Scale bar: 10 μm); (**B**) The porosity percentage of collagen hydrogel scaffolds in different collagen concentrations, including 4 mg/mL, 5 mg/mL, 6 mg/mL, and 7 mg/mL.

FE-SEM images of acellular hydrogel (Figure 4) showed the formation of a three-dimensional high porous structure of the collagen hydrogel with uniform network connections. Evaluation of FE-SEM images after OE-MSCs encapsulated in the collagen hydrogels (Figure 4A) showed that the human OE-MSCs were alive and elongated with attached adequately to the hydrogels in all groups. Figure 4B indicates the porosity of collagen hydrogel scaffolds in different concentrations of 4 mg/mL, 5 mg/mL, 6 mg/mL, and 7 mg/mL. The results obtained in this study show that all scaffolds are very porous and above 90% porosity. As shown in Figure 4B, the percentage of porosity was associated with collagen concentration. The porosity of scaffolds containing collagen 4 mg/mL is higher than other scaffolds, and there is no significant difference between groups.

### 3.5. Collagen Concentration of 7 mg/mL Has a Storage Modulus Comparable to the Other Concentrations

**Figure 5:**
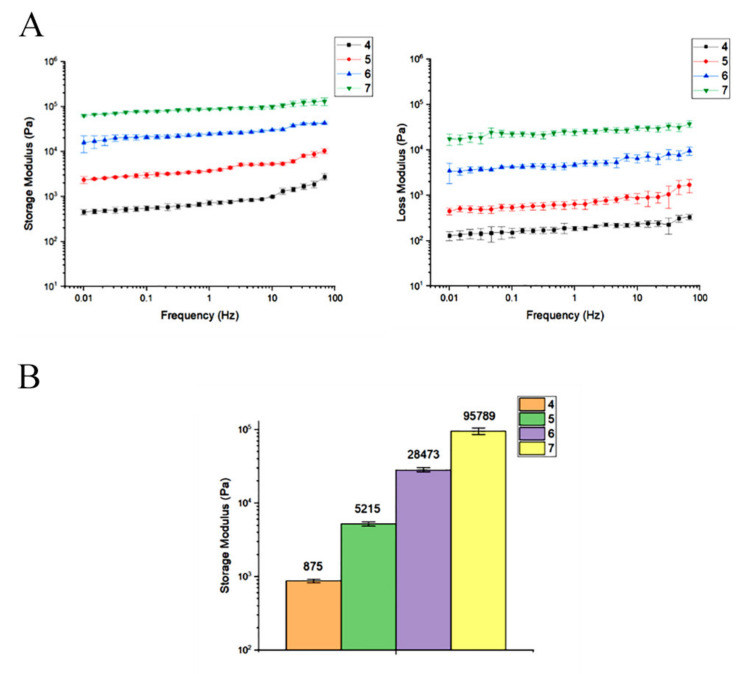
(**A**) Rheological evaluation of storage and loss modulus of different collagen hydrogel concentrations during frequency sweep study. (**B**) At 6 Hz, the effect of collagen concentration on hydrogel storage modulus (G′).

The mechanical properties include a rheological assessment characterized storage modulus (G′) and loss modulus (G″) vs. Frequency of hydrogels with different concentration of collagen (4 mg/mL, 5 mg/mL, 6 mg/mL, and 7 mg/mL) during frequency sweep analysis. G′ was better than G″, with minimal frequency dependency in all groups, according to the examined data presented in Figure 5. Rheological data analysis showed that the storage modulus was associated with collagen concentration significantly enhanced from ~875 Pa in group contained 4 mg/mL collagen to ~95,789 Pa in the group had 7 mg/mL collagen at 6 Hz.

### 3.6. The Increase in the Concentration of Collagen Hydrogel Improved Osteogenic Differentiation of OE-MSCs

**Figure 6:**
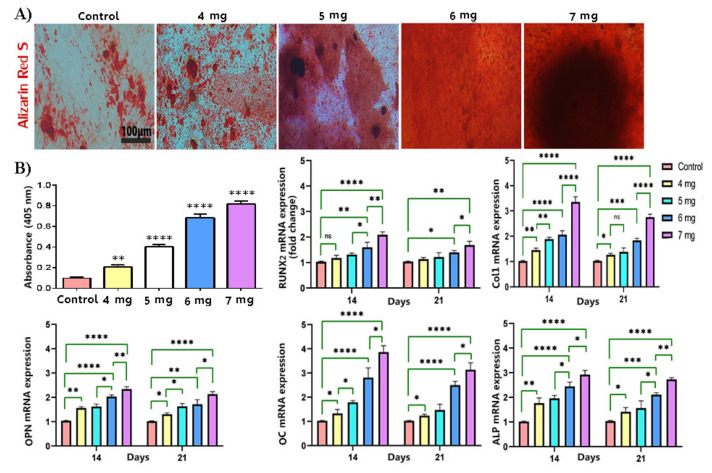
Osteogenic differentiation of OE-MSCs. (**A**) The extracellular matrix mineralization evaluation of encapsulated OE-MSCs in different concentration of collagen; The extracellular matrix mineralization was stained by Alizarin red S; quantifying the level of calcium production by Alizarin red S; (**B**) Osteogenic-related gene expression. Relative osteogenic marker expression of Runx2, Col1, OPN, OC, and ALP (*n* = 3, * p < 0.05; ** p < 0.01; *** p < 0.001; **** p < 0.0001), (scale bar: 100 μm).

The ECM mineralization was evaluated on day 21 by Alizarin Red S staining. All data were assessed compared to two dimensional cultured of human OE-MSCs exposed to the osteogenic differentiation media as a control group. According to the data (Figure 6), the OE-MSCs encapsulated in all collagen concentrations showed calcium deposition. Additionally, the human OE-MSCs encapsulated in the collagen 7 mg/mL showed the highest calcium deposition than other groups. A significant difference was demonstrated between collagen 7 mg/mL absorbance and other groups (Figure 6B).

The real-time PCR was carried out to evaluate the osteogenic differentiation potency of encapsulated OE-MSCs in various concentrations to evaluate the osteogenic differentiation potency of encapsulated OE-MSCs in different concentrations of collagen hydrogel. Table 1 lists the gene primer sequences used in real-time PCR. The cycle threshold technique was used to approximate gene expression in relation to β-actin expression. The gene expression of significant osteogenic markers included Runx2, Col1, OPN, OC, and ALP, was evaluated on days 14 and 21. According to the data (Figure 6B), the up-regulation of osteogenic markers was shown in all groups during 21 days compared to the control group. These data demonstrate that the osteogenic differentiation of OE-MSCs was done in all groups, but the expression level of osteogenic markers was significantly higher on days 14. The level of gene expression was associated with collagen concentration, and the highest gene expression was obtained for encapsulated OE-MSCs in collagen 7 mg/mL in all genes. Furthermore, the significant difference between collagen 7 mg/mL and 6 mg/mL was shown in all groups.

### 3.7. Encapsulated DiI-Labeled Human OE-MSCs in Collagen Hydrogel Were Traced in Rat Defection

**Figure 7:**
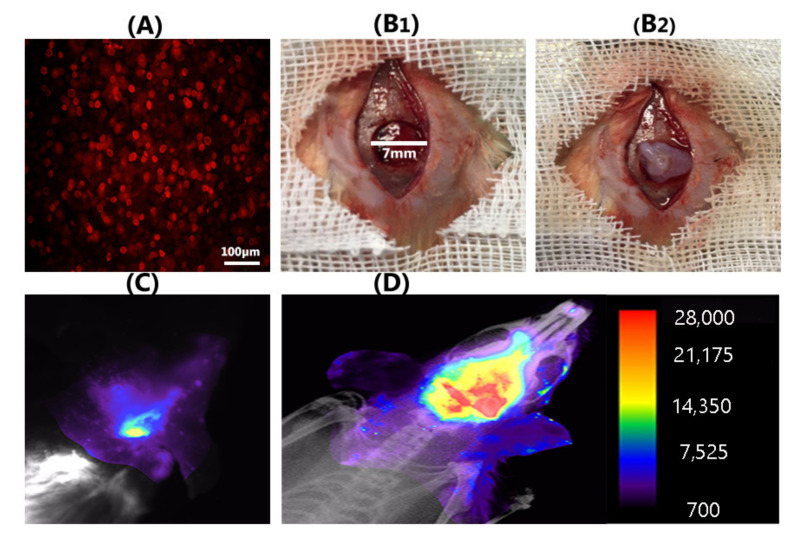
Human OE-MSCs labeled with DiI (**A**); and implanted with collagen hydrogel in a rat calvarial defection with critical size defect (7 mm) (**B1**,**B2**); Representative optical image of blank scaffold group (**C**); and covering images of X-ray and fluorescence images reveal a large number of DiI^+^ OE-MSCs at the calvarial defective region in OE-MSCs group after 72 h (**D**) (scale bar: 100 μm).

For cell tracking in the defection region, 1 × 10^6^ cells/mL of human OE-MSCs were labeled with DiI (Figure 7A). Next, these stem cells encapsulated in 7 mg/mL collagen hydrogel were implanted in the rat calvaria defection with a 7 mm critical size defect (Figure 7(B1,B2)). In vivo monitoring of alive DiI positive OE-MSCs in the lesion of calvaria was performed using optical imaging modality. Based on the data, we observed alive human OE-MSCs in the lesion area after 72 h (Figure 7D); conversely, in the blank scaffold group (OE-MSCs free), we did not recognize any trace of cell presence (Figure 7C).

### 3.8. The Highest Bone Healing Rate of Rat Calvarial Defection Observed in the OE-Mscs Group

**Figure 8:**
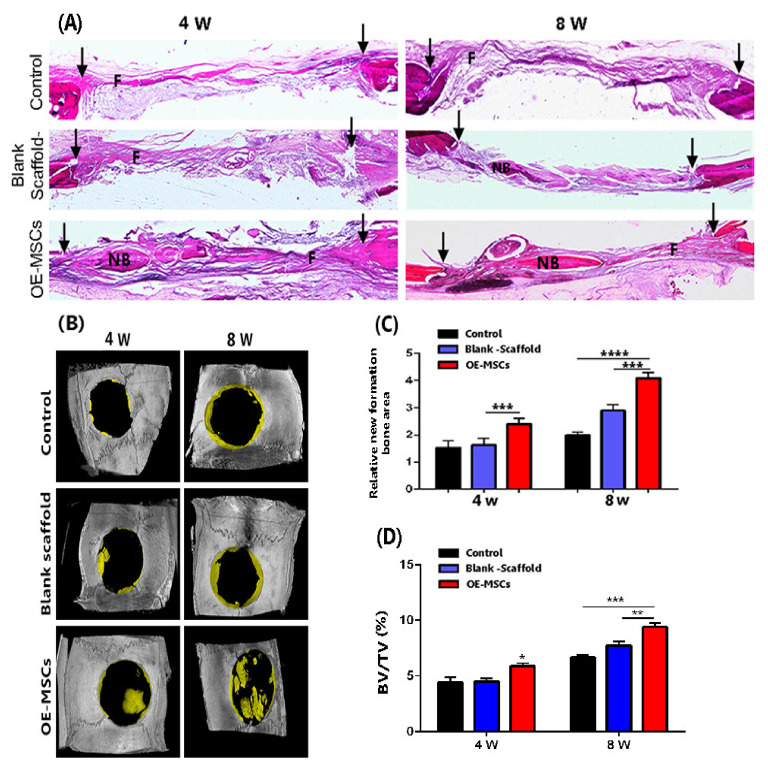
(**A**) After 4 and 8 weeks of implantation, H&E staining images showed new bone development in the calvarial defect area; (**B**) Representative micro-CT images of rat calvarial defects treated with or without OE-MSCs after 4 and 8 weeks; (**C**) The relative areas of fresh bone formation in three experimental groups, as determined by histological examination, (*n* = 5, * p < 0.05; ** p < 0.01; *** p < 0.001; **** p < 0.0001). Human OE-MSCs encapsulated in 7 mg/mL collagen hydrogel regenerate calvarial bone in vivo; (**D**) In rat calvarial defect models, summarized data showing new bone tissue volume/total defect volume (BV/TV) for newly developed bone tissue.

After four weeks of implantation, H&E staining showed that the collagen hydrogel had vanished, but fibrous tissue defect had been filled (Figure 8A). Freshly formed bone tissues [15] were also evident in the OE-MSCs classes. At both 4 and 8 weeks, statistical analysis showed that the OE-MSCs group developed more NB in the calvarial defect area than the other groups. Compared to the other groups, the relative region of fresh bone tissue in the OE-MSCs population improved significantly over time in 8 weeks (Figure 8A,B). Micro-CT images also confirmed the new bone generation in the experimental groups in the rat calvarial defection (Figure 8B). The micro-CT images exhibited that the control and blank scaffold group revealed minimal bone healing (less than 5%) than OE-MSCs groups at four weeks. We also detected the highest rate of new bone formation in the OE-MSCs group than other groups after eight weeks. The region of bone growth was calculated by dividing the total defect volume by the new bone volume. In comparison, the group treated with OE-MSCs exhibited a significantly higher percentage of healing defects (about 10%) at eight weeks, associated with the osteogenic differentiation of implanted OE-MSCs that was encapsulated in collagen hydrogel (Figure 8D).

## 4. Discussion

This proof-of-concept study proposed a natural-based scaffold consisted of olfactory-derived MSCs for bone regeneration. The data from this study demonstrated that the encapsulated stem cells within collagen hydrogel cultured in osteogenic media could differentiate to osteoblasts and accelerate bone regeneration post-implantation.

Some requirements, such as biocompatibility and degradability of materials and osteoprogenitor cells, should be addressed when designing bone tissue engineering. Cell-loaded scaffolds presented an attractive option for bone formation in critical and massive defects. Additionally, there is growing interest in identifying easy available with high proliferative and osteogenic properties cell sources for bone tissue engineering. Therefore, collagen hydrogels were developed to create a supportive carrier for our study.

In 2009, Delmore et al. [14] reported a new type of undifferentiated cells with a neurogenic and osteogenic property from the olfactory mucosa defined as OE-MSCs. Due to their easy access, higher mitotic activity, and proliferation rate than bone marrow and Wharton jelly MSCs, they have received more attention in recent years. Since these multipotent stem cells have telomere adjustment and maintain telomerase activity, they can sustain proliferative and culture ability for the long term even more than 15 weeks [2]. In other adult stem cells, the telomeres are decreased, and telomerase gradually loses its activity [23]. As expected, the morphology and characteristics of surface markers of isolated cells were like mesenchymal and ectodermal cells.

The collagen type I, which is pure and unchanged, was extracted from the rat tail. The FTIR spectrum was performed to detect the functional groups in the structure of extracted collagen. Moreover, it shows unique molecular vibrations, including amide A, amide B, amide I, amide II, and amide III. Carbonyl groups (C=O) were identified as amide I at peak 1626.48 cm^−1^, indicating the presence of secondary structure in collagen proteins. The peak found at 1535.60 cm^−1^ indicated the tension of N-H and C-N that revealed amide II. Amide III was found at peak 1232.42 cm^−1^ and indicated the existence of C-N that confirm the presence of proline [24]. This evidence follows the data reported on similar research that has been effectively extracted collagen type I from adult tissues [25]. SDS-PAGE is a valid analysis to determine the type of collagen. Collagen type I has a striking pattern in terms of migration on an electrophoresis gel. After it runs on, the gel should be separated by α-monomer chains and β-dimer chains. The intensity and molecular weight of α_2_ chain are lighter than α_1_ because the collagen I triple helix is made up of two chains (α_1_ and α_2_). In this study, the molecular weight for α_1_, α_2_ was, respectively, about 125 kDa, and 110 kDa and β-chain was higher than 170 kDa, and the α_1_ bond was heavier than the α_2_ band. These data showed that the extracted collagen has a molecular weight similar to related published articles [26].

Collagen is a natural polymer in medical application due to high biocompatibility, suitable biodegradability, and low immunogenicity for cell encapsulation [27]. Collagen fibrils have some cell-binding sites, such as Arg-Gly-Asp (RGD) sequence, which interact and connect to cells. They significantly facilitate the attachment of cells to the hydrogel [28]. Mazzoni et al. [29], confirmed that collagen/hydroxyapatite is an ideal microenvironment for MSCs, with increased cell binding, cell augmentation, matrix synthesis, and mineralization to that shown in osteogenic condition media. The stimulation of distinct osteogenesis signaling pathways, such as extracellular signal-regulated protein kinase is mediated via collagen fibers [30].

For this reason, we investigated collagen type I hydrogel with different collagen concentrations based on some criteria, including gelation time, mechanical strength near to bone tissue and natural effect on cell attachment and proliferation.

The rheological test confirms that collagen hydrogel with 7 mg/mL concentration has optimum viscoelastic behavior. A frequency sweep was used to analyze the storage modulus (G′) and loss modulus (G″) of hydrogels with varying collagen concentrations. According to the obtained data, G′ was higher than G″ in all groups. No intersection showed the viscoelasticity and gelation rate and stability of all groups, and the storage modulus was related to collagen concentration. The viscoelasticity and gelation rate depended on collagen concentration, and the highest viscoelasticity was observed in the group containing 7 mg/mL collagen. The rheology analysis showed that collagen hydrogel storage modulus in concentrate of 7 mg/mL was close to the native bone (100 kpa) [16]. Collagen hydrogels, in particular, maintain the viability of encapsulated human OE-MSCs during in vitro tests such as live/dead and resazurin assays. The results have shown that the biocompatibility of collagen hydrogels was appropriate in all collagen concentrations. As proven in previous studies, the high concentration of collagen in the scaffold structure increases the proliferation rate of cells [31]. Therefore, this carrier provides an outstanding opportunity for cell delivery into the damaged tissue. Encapsulated human OE-MSCs maintain their morphology and live over culture time. FE-SEM images also demonstrated the cell attachment ability and the morphology of OE-MSCs on the surface of the hydrogel. As shown in Figure 4, OE-MSCs were alive and elongated with correct attachment to the hydrogels in all groups. Furthermore, the porosity of 90% is required to facilitate the flow of nutrients and metabolism and create a suitable space for the reconstruction of scaffolds [32].

In this study, collagen hydrogels’ porosity containing different collagen concentrations was between 94.5–97%. The percentage of porosity was dependent on collagen concentration, and the group with 7 mg/mL collagen had lower porosity than other groups. In both classes, Alizarin red staining and real-time PCR data were used to show the osteogenic differentiation of human OE-MSCs, but gene expression was significantly better with increasing collagen concentration. The expression of RUNX2 and OPN plays a vital role in bone differentiation and formation. Additionally, Col-I and ALP genes expression are essential in the early phases of ossification and OCN in the middle and late ossification phases [33,34].

According to this study results, the 7 mg/mL collagen concentration was associated with up-regulation of ECM mineralization and the highest osteogenic gene expression of encapsulated human OE-MSCs. High regulation of osteogenic markers in 7 mg/mL collagen groups indicates this scaffold showed considerable ability to promote osteoblast proliferation and differentiation.

Optical imaging results showed that OE-MSCs labeled with DiI, which were encapsulated in collagen 7 mg/mL, could survive at the rat calvaria defection site for up to 72 h after transplantation are traceable.

Micro-CT analysis of human OE-MSCs encapsulated in 7 mg/mL collagen hydrogel in rats calvarial defects that were certified to promote bone regeneration. This group has a significant impact on bone formation after 4 and 8 weeks. Considering histological evidence and micro-CT results, bone defect in the OE-MSCs-treated rats is significantly covered by new bone compared to other groups. The most important observation from the data is that osteoblast proliferation and bone growth are induced at the defect’s margins.

The previous study reports potential alternatives of bone marrow MSC-encapsulated collagen microspheres that can be differentiated into osteogenic and chondrogenic lineages in 3D collagen microspheres [35]. Collagen microspheres with committed MSCs possess osteoinductive-activities as confirmed by the ability to induce undifferentiated MSCs to commit to the osteogenic lineage [36]. We hypothesize that encapsulated human OE-MSCs in collagen hydrogel with osteoinductivity capacity have a good alternative candidate for fresh bone grafting in tissue defection

Based on our results obtained from bone tissue regeneration, it can be declared that OE-MSCs lead to increasing osteogenesis in the defection site. This event can occur on two factors: (1) high proliferation rate and osteogenic differentiation of OE-MSCs encapsulated in collagen 7 mg/mL that was proved in vitro part; (2) OE-MSCs osteogenesis secretome can exert significant paracrine effects in the surrounding tissue defection [3]. These paracrine signals can promote osteoblast cells’ survival in defection or recruit osteoblast progenitor cells from the other organs and improve bone regeneration.

## 5. Conclusions

A collagen-based hydrogel for human OE-MSCs delivery that allows highly efficient cell transplantation to the bone defect was fabricated and characterized. We proposed an optimum collagen concentration that meets the criteria of being a feasible carrier, biocompatible, and biodegradable. This construct provides human OE-MSCs survival and osteogenic differentiation and augmentation in vitro and shows mineralization and bone formation in vivo.

## Figures and Tables

**Table 1:** Primer sequences for real-time RT-PCR.

Gene Name	Primer
RUNX2	F GCCTCCAAGGTGGTAGCCC R CGTTACCCGCCATGAGAGTA
Collagen I (Col1)	F TCCGACCTCTCTCCTCTGAA R GAGTGGGGTTATGGAGGGAT
Osteopontin (OPN)	F GACCTGACATCCAGTACCC R GTTTCAGCACTCTGGTCATC
Osteocalcin (OC)	F GCAAAGGTGCAGCCTTTGTG RGGCTCCCAGCCATTGATACAG
Alkaline Phosphatase (ALP)	F GCACCTGCCTTACTAACTC RAGACACCCATCCCATCTC
Β-actin	F CTTCCTTCCTGGGCATG R GTC TTTGCGGATGTCCAC

## Data Availability

Data sharing is not permitted. This analysis did not generate or review any new data. This report does not include data sharing.
